# Loss by Typhoid Fever

**Published:** 1897-11

**Authors:** 


PROF. W. P. MASON, of Troy, estimates that the loss to Philadelphia by typhoid fever amounts in value to an annual tax of $1,392,000. This loss, he claims, could be easily prevented.

